# Poor Agreement between Responses to the International Physical Activity Questionnaire and Objective ActiGraph^®^ Data among Persons with Major Depressive or Bipolar Disorders

**DOI:** 10.3390/ijerph192214913

**Published:** 2022-11-12

**Authors:** Rafael Bonfim do Nascimento, Rafael Pereira Guimarães Santos, Tabatah Hellen Santos Gomes, Carolina Nunes França, Fabricio Eduardo Rossi, Decio Gilberto Natrielli-Filho, José Claudio Jambassi-Filho, Saulo Gil, Brendon Stubbs, Beny Lafer, Lucas Melo Neves

**Affiliations:** 1 Post-Graduation Program in Health Sciences, Santo Amaro University, São Paulo 04743-030, Brazil; 2 Immunometabolism of Skeletal Muscle and Exercise Research Group, Department of Physical Education, Federal University of Piauí (UFPI), Teresina 64049-550, Brazil; 3 Professor at Graduate Program in Science and Health, Federal University of Piauí (UFPI), Teresina 64049-550, Brazil; 4 Residency Specialty in Psychiatry, Santo Amaro University, Sao Paulo 04743-030, Brazil; 5 Department of Physical Education, Padre Albino University Center, Catanduva/SP, Sao Paulo 15806-310, Brazil; 6 Graduation Physical Education, Santo Amaro University, Sao Paulo 04743-030, Brazil; 7 Department of Psychological Medicine, Institute of Psychiatry, Psychology and Neuroscience, King’s College London, London WC2R 2LS, UK; 8 Bipolar Disorder Program (PROMAN), Department of Psychiatry, University of São Paulo Medical School, Sao Paulo 14049-900, Brazil

**Keywords:** mental health, physical activity assessment, accelerometry, exercise, measurement

## Abstract

The purpose of this research was to investigate the degree of agreement between data from the International Physical Activity Questionnaire—Short Form (IPAQ) and accelerometer (ActiGraph^®^) readings for physical activity (PA), classified as moderate, vigorous, and moderate–vigorous PA, and sedentary behavior (SB) in participants with major depressive or bipolar disorder. Following a cross-sectional observational design (*n* = 30), participants used an accelerometer for 4 to 7 days (minimum of 10 h per day) and answered the IPAQ (for the same period as accelerometer use). Our results suggest significant differences (*p* < 0.05) when comparing the ActiGraph^®^ and IPAQ data: for moderate PA, 155 min vs. 25 min per week; for moderate–vigorous PA, 157 min vs. 50 min per week; and for SB, 8 h vs. 3 h per day. Spearman’s correlation coefficients (ActiGraph^®^ and IPAQ) were low for moderate PA, vigorous PA, and moderate–vigorous PA (rho = 0.03 to 0.13). The Bland–Altman plot showed a bias of −75 min for moderate PA, 9 min for vigorous PA, −66 min for moderate–vigorous PA, and −5 h for SB. Considering the differences observed and the objectivity of the ActiGraph^®^ measurements, whenever possible, we recommend ActiGraph^®^ measurements of PA and SB for these clinical groups.

## 1. Introduction

Major depressive disorder (MDD) is characterized by several episodes of sadness, loss of interest, changes in sleep, hopelessness, irritability, anhedonia, psychomotor agitation or lethargy, decreased appetite, fatigue, and suicidal ideation and/or behavior [1]. Bipolar disorder (BD) is characterized by mood fluctuations between depressive and manic symptoms (persistent restlessness and/or exaltation for more than a week, accompanied by acute symptoms such as inflamed self-esteem, reduced sleep, varied speech, accelerated and exacerbated thoughts, and risky behaviors) or recurrent hypomania (a phase during which manic symptoms are decreased) [1].

Both MDD and BD are mood disorders that, together, affect more than 400 million people [2]. In general, people with MDD or BD have a 1.4 to 2.0 times greater risk of presenting non-communicable chronic diseases (e.g., obesity, diabetes, and cardiovascular disease) compared to the general population [3]. This increased prevalence/risk of physical comorbidities partially explains the higher mortality rate in people with MDD or BD [4]. In the context of higher risk factors for chronic diseases and mortality in these populations, it is critically important for researchers to aid health professionals in designing strategies to mitigate non-communicable chronic diseases in this population [3].

In this regard, physical activity (PA)—energy expenditure above resting levels due to muscle activity, ≌ more than 1.5 metabolic equivalents (METS) [5]—is a potentially modifiable behavioral indicator of health status in the general population, and a lower time spent in PA is an independent predictor of lower physical and mental health among people with MDD or BD [6,7,8,9,10,11,12,13,14]. At the other end of the activity spectrum, there is increasing evidence to suggest that sedentary behavior (SB)—reduced energy expenditure below ≌ 1.5 METS in positions such as sitting, reclining, or lying down [15]—may be associated with a range of physical comorbidities [16] and worsened mental health [17,18]. Previous research identified that people with MDD or BD spend little time in moderate–vigorous intensity PA (MVPA) and a lot of time in SB, compared with healthy people [19]. This relative trend toward less PA and more SB for persons with these mental disorders clearly places them at risk for greater health problems and diminished physical wellbeing. 

Researchers generally use one of two main data collection methods for capturing PA and SB data. The first is through self-report, using standardized questionnaires, such as the International Physical Activity Questionnaire (IPAQ) [20]. Although these PA and SB assessment tools have been extensively employed in clinical practice and research due to their ease of use and relatively low cost [21], they are subjective and can present measurement errors [22]. Furthermore, the personal characteristics of the respondents appear to influence the accuracy of their self-report questionnaires [23], partly due to poor memory, which may be associated with depressive conditions [24]. A second common way to capture PA and SB data is through an objective assessment, using movement measurement devices, such as the ActiGraph^®^. This device is highly accurate for monitoring PA and SB in environments without movement obstruction [25]. However, these approaches require monitoring for consecutive days (typically seven) [26], are expensive, and can be challenging in clinical and research settings. 

There is some preliminary evidence that healthy groups, for example, Asian women [27], groups with specific diseases, such as autoimmune diseases [28], and even populations with mental disorders [29,30] demonstrate the same trend: poor agreement (PA time underestimated by IPAQ) between the subjective and objective methods. Understanding the agreement between these measurement methods in people with MDD or BD could aid future researchers to choose accurate assessment methods in order to better investigate measures of PA and SB. In addition, if the results show a poor correlation between the IPAQ and accelerometer, healthcare professionals can select alternative instruments (other questionnaires) for measuring PA and SB in MDD or BD.

We expected that the analysis of these data gathering methods for patients with MDD or BD would demonstrate a similar significant difference, with direct relevance for the accuracy of data that health professionals may rely upon in their intervention efforts for these populations. Therefore, our objective, in the current study, was to analyze the degree of agreement between these common methods of measuring PA and SB in participants with MDD or BD. We hypothesized that there would be a poor level of agreement between subjective (IPAQ) and objective (ActiGraph^®^) measurements of PA and SB, with the IPAQ data underestimating PA and SB in relation to ActiGraph^®^.

## 2. Materials and Methods

### 2.1. Study Design and Participants

Using a cross-sectional, observational design, in this study, we included participants diagnosed with either MDD or BD (according to the Mini International Neuropsychiatric Interview (MINI)) [31,32] who were under treatment in a psychiatric outpatient clinic. Our local ethics committee approved the study (protocol number 3,655,943) and all participants gave their informed consent to participate in this research. The research was carried out following the principles set forth in the declaration of Helsinki [33]. Participants were recruited at the outpatient clinic using posters and pamphlets, through invitations from the treating psychiatric residents during their consultations with the patients or through spontaneous approaches toward the patients from the researchers. If registered patients did not have scheduled consultations during the period of this research, we contacted them by telephone to explain the study and invite their voluntary participation. 

The study inclusion criteria were that participants (a) were aged between 18 and 60 years; (b) without physical disability; (c) without musculoskeletal injuries; and (d) diagnosed with MDD or BD from the psychiatry team at the clinic where the research was conducted, with confirmation of this diagnosis through a formal psychiatric interview [31,32]. Exclusion criteria were that participants (a) were unable or unwilling to provide informed consent for the study; and/or (b) presented another comorbid mental illness during the confirmation of their MDD or BD (e.g., schizophrenia, substance-abuse disorder, etc.).

After confirmation of the diagnosis using the MINI, another interview was performed to characterize the participant (e.g., with respect to age, education, drugs used to treat MDD or BD, etc.). Additionally, the participants’ depressive symptoms (MDD or BD) were measured using the Montgomery–Asberg Depression Scale (MADRS) [34], and their manic symptoms (BD) were measured by the Young Mania Rating Scale (YMRS) [35]. Finally, we assessed the time the participants were engaged in PA and SB through the use of an ActiGraph^®^ (model GT9X, Pensacola, FL, USA) for seven days and by asking them to self-report their PA and SB on the IPAQ [20] over the same seven-day period as the ActiGraph^®^ use.

### 2.2. Interviews and Tools for Characterizing the Participants 

We applied a seven-part questionnaire to gather participant information regarding (a) general information: name, email, gender, contacts, age, profession, and date of birth; (b) educational history: elementary school, high school, or university education; (c) history of comorbidities: questions about the presence of diseases such as hypertension, diabetes mellitus, musculoskeletal disorders, obesity, or any other diseases that were being treated; (d) number of drugs used to treat their psychiatric disorders (MDD or BD); (e) anthropometric measurements, including recording of weight, height, and body mass index (BMI); and (f) questions on subjective health perceptions.

### 2.3. Mini International Neuropsychiatric Interview (MINI) 

As noted above, we also used the MINI to screen patients for symptoms of MDD and BD. The MINI is a structured diagnostic interview questionnaire to evaluate the psychiatric disorders described within the Diagnostic and Statistical Manual of Mental Disorders (DSM-V) and the International Classification of Diseases (ICD-10) [32]. The MINI is a brief tool (20–30 min to apply), which is especially convenient for diagnosing psychiatric patients in daily clinical practice. The questions contained in the questionnaire are formulated to allow only ‘yes’ or ‘no’ answers. The MINI explores all the inclusion and exclusion criteria and the chronology for the 23 diagnostic categories of the DSM-V, and it has demonstrated satisfactory reliability for all diagnostic sections [32]. 

To confirm the diagnosis of MDD, positive answers to questions A1 (A or B) and A2 (A or B) show a possibility of disease. In the case of affirmative answers to questions A1 or A2, seven additional questions are asked (A3; (A) appetite; (B) difficulty sleeping; (C) moving slower than usual; (D) feeling tired; (E) feeling worthless or guilty; (F) difficulty concentrating; (G) repeatedly thinking about death). If responses to five of these seven questions are positive (in A1–A3), the patient is deemed to have a confirmed diagnosis of MDD [32].

To confirm the diagnosis of BD, the MINI assesses both manic and hypomanic episodes. Positive answers to questions C1 (A or B) and C2 (A or B) show a possibility of the disease. In the case of affirmative answers to questions C1 or C2, seven additional questions are asked (C3; (A) euphoria; (B) difficulty sleeping; (C) fast speech; (D) fast thinking; (E) distraction; (F) increased intensity of daily activities; (G) feelings of recklessness or irresponsibility). If five of these seven questions are positive (C3), the periods of manic and hypomanic episodes are evaluated, confirming the main symptoms of BD [32].

### 2.4. Montgomery–Asberg Depression Scale (MADRS)

We used the MADRS scale [34] to determine the presence and degree of depressive symptoms. The MADRS is comprised of ten items (sadness, inner tension, decreased sleep, reduced appetite, distraction, lassitude, anhedonia, pessimism, and suicidal ideation). Nine items are based on the patient’s self-reporting and one is based on clinical observations. Scores range from 0 to 60; the higher the score, the greater the likely presence of depressive symptoms [34]. The MADRS has been found to be valid for this purpose [35], with good internal consistency (Cronbach’s alpha = 0.91) and reliability (intraclass correlation coefficient = 0.87) [35]. 

### 2.5. Young Mania Rating Scale (YMRS)

We used the YRMS [36] to quantify the presence of manic symptoms. The YRMS is composed of 11 items (high mood, motor agitation, disproportionate sexual interest, sleep disorders, irritability, speech rate, language disorder, speech content, aggressiveness, appearance (clothing), and insight). Each item consists of five explicitly defined severity levels. Severity ratings are based on the patient’s subjective report of their clinical condition over the previous 48 h and the investigator’s observations during the interview. The YMRS scores range from 0 to 60; the higher the score, the greater the presence of manic symptoms [36]. The YRMS is a valid instrument with good internal consistency (Cronbach’s alpha = 0.67) and reliability (intraclass correlation coefficient = 0.97) [37].

### 2.6. International Physical Activity Questionnaire Short Form (IPAQ)

The IPAQ [20] is a self-reporting instrument comprised of eight questions regarding waking time (2 questions), moderate PA (MPA) (2 questions), and vigorous PA (VPA) (the sum of MPA and VPA = moderate and vigorous PA—MVPA) performed during the previous seven days. In addition, time spent sitting (2 questions) is evaluated as a separate question and is used as an indicator of weekday and weekend SB. 

To calculate the IPAQ score, the time was first converted from hours and minutes to minutes. Second, the number of days of walking, moderate or vigorous intensity PA, and SB were multiplied by the time in minutes to calculate the walking time, moderate or vigorous intensity PA, and SB. For moderate–vigorous (MVPA) calculations, the times of both intensities were added together.

### 2.7. ActiGraph^®^

The ActiGraph^®^ accelerometer is an electronic device capable of measuring body movement or acceleration in three planes: anteroposterior, mediolateral, and vertical. In addition, this device quantifies the frequency, duration, and intensity of these movements, as well as the frequency and duration of amount of time spent sitting, lying, and reclining, which we call SB, as defined by the Terminology Consensus Project of Sedentary Behavior Research Network (SBRN) [15]. Among the measures provided by the ActiGraph^®^, we used the time measures of light PA (LPA), MPA, VPA, MVPA, and SB.

We used the ActiGraph^®^ (model GT9X, Pensacola, FL, USA) in this research. Collection of data, wear time validation, and scoring are the three principal actions necessary for measuring PA and SB with the ActiGraph^®^. For data collection, the participant is required to wear the ActiGraph^®^ for seven days, fixed with an elastic strap (right side of the waist), for at least 600 min of daily use, removing it only when initiating nighttime sleep and coming into direct contact with water [38]. To analyze the data and determine wear time validation, after wearing and returning the ActiGraph^®^, we used the Actilife software (ActiLife, v6.13.4, Pensacola, CA, USA), using “Choi 2011” parameters (zero-count threshold during a non-wear time interval; 60-min time window for consecutive zero or non-zero counts; and an allowance of a 2-min interval of non-zero counts with the upstream or downstream 30-min consecutive zero-count window for detection of movement artifacts) [39]. The validation considered at least four days of use (one weekend day and three weekdays) [38,40]. We calculated the final score using Actilife software, adopting the suggested cut-off point for adults as there are no specific cut-off points for people with MDD or BD. We followed the parameters of Freedson (2011) [40] to classify SB (0–199 counts), light PA (200–2686 counts), moderate PA (2687–6166 counts), and vigorous PA (>6166 counts). We used the time in each of these counts (minutes per day) as a measure. In case of use between four and six days, we calculated an estimate of activity during the day or days not used; e.g., if the participants used the device on six days for 600 min or more, and the total time of MVPA was 180 min, we calculated 30 min per day of MVPA. In this way, we estimated one day without data, using the mean of the other days. Thus, the value in this example was 30 min of MVPA per day, giving an estimated 210 min of MVPA in seven days.

### 2.8. Statistical Analyses 

All analyses were performed using the software package Statistical Package for the Social Sciences (SPSS, version 20.0, IBM, Corp. Chicago, IL, USA). We adopted a statistical significance level of *p* ≤ 0.05. We utilized the Shapiro–Wilk and Levene tests to determine the normality of the data distribution and equality of variance, respectively. The data presented a non-parametric distribution (MPA, VPA, MVPA, and SB), and we used the Mann–Whitney test to compare the data obtained by the IPAQ and ActiGraph^®^ (in the MDD and BD samples separately and in the pooled MDD and BD samples). The data are presented as medians and interquartile ranges. 

We calculated Spearman’s correlation coefficients only for pooled data (MDD and BD together) to test for the possible associations between PA and SB engagement times across the two instruments. The interpretation of Spearman’s correlation coefficients was as follows: 0 to 0.39—weak; 0.40 to 0.69—moderate; >0.70—strong [41]. 

Finally, we performed a Bland–Altman plot analysis [42,43], but only for both groups together (pooled—MDD and BD), to assess the agreement between the data obtained by the IPAQ and ActiGraph^®^. The x-axis (average of measurements obtained by IPAQ and ActiGraph^®^) and y-axis (difference between measurements obtained by IPAQ and ActiGraph^®^), bias, standard deviation (SD), and 95% confidence interval (CI) of the difference are presented as a linear regression (with an equation).

## 3. Results

**Figure 1:**
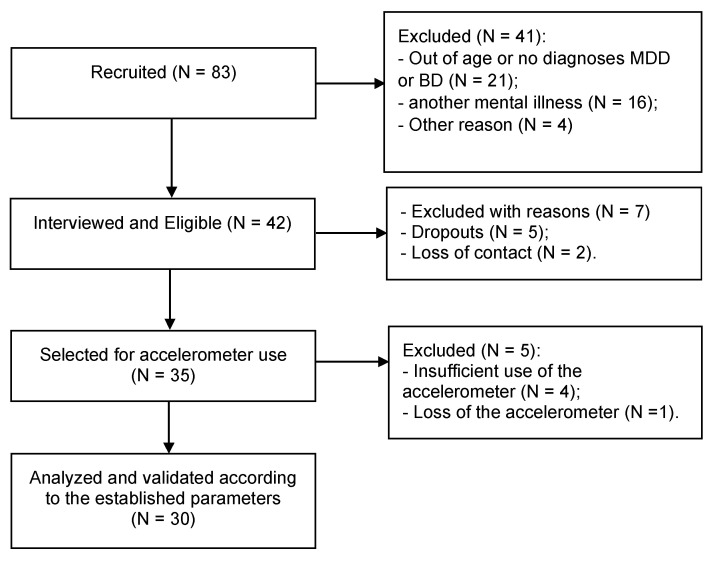
Study flowchart.

Of the 83 prospective participants approached, 41 were excluded, for the reasons detailed below. Of the remaining 42 participants interviewed, 30 participants continued until the end of the research and their data were validated according to the required parameters, as shown in Figure 1.

Table 1 details the characteristics of the sample included in the study. Briefly, the sample consisted of predominantly females (83%) of middle-age (M = 42.5, SD = 10.8 years). Regarding symptoms, the participants presented with moderate symptoms of depression and no mania states. Obesity (MDD: 61%; BD: 25%) and hypertension (MDD: 22%; BD: 25%) were more prevalent comorbidities in MDD than BD. Data from all participants that used the ActiGraph^®^ for 4 to 7 days were included. The average number of days using the ActiGraph^®^ was (a) MDD: 6.1, SD = 1.1 days; and (b) BD: 6.1, SD = 1.0 days, totaling 186 days (or 88.6% of possible days). Thus, we estimated ActiGraph^®^ data for 24 days (11.4%; 14 days for patients with MDD and 10 days for patients with BD).

### 3.1. Comparisons between the ActiGraph^®^ and IPAQ Data

Table 2 shows the measurements obtained by the IPAQ (walking, MPA, VPA, MVPA, and SB) and ActiGraph^®^ (LPA, MPA, VPA, MVPA, and SB) of participants with MDD, BD, and both together (pooled—MDD and BD). 

In the comparisons between the IPAQ and ActiGraph^®^ (Table 2), there were statistically significant differences (*p* < 0.05) (time underestimated by IPAQ) for patients with MDD (MPA, MVPA, and SB), patients with BD (MPA and SB), and for both groups of patients pooled together (MPA, MVPA, and SB).

### 3.2. Associations between Data from the ActiGraph^®^ and IPAQ

**Figure 2:**
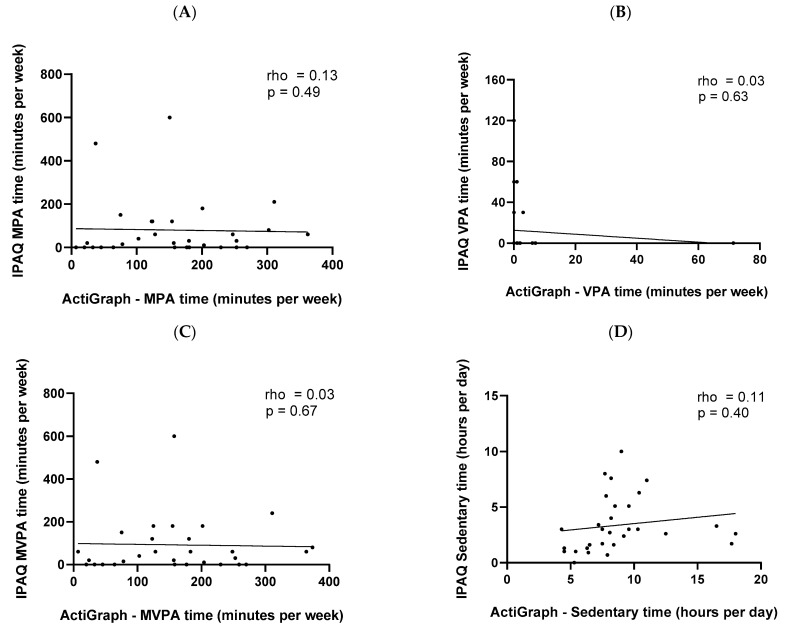
Associations between the IPAQ and ActiGraph^®^ (Spearman correlation) measures regarding major depressive disorder or bipolar disorder: for MPA—Panel (**A**); VPA—Panel (**B**); MVPA—Panel (**C**); sedentary behavior—Panel (**D**); rho = Spearman’s Correlation Coefficient; MPA = moderate physical activity; VPA = vigorous physical activity; MVPA = moderate and vigorous physical activity.

Figure 2 presents data correlations for data gathered via the IPAQ and ActiGraph^®^ for MPA, VPA, MVPA, and SB (pooled—MDD and BD together). 

The associations between the IPAQ and ActiGraph^®^ were weak and non-significant for MPA (Panel A; rho = 0.13, *p* = 0.49), VPA (Panel B; rho = 0.03, *p* = 0.63), MVPA (Panel C; rho = 0.03, *p* = 0.67), and SB (Panel D; rho = 0.11, *p* = 0.40).

### 3.3. Bland–Altman Plot

**Figure 3:**
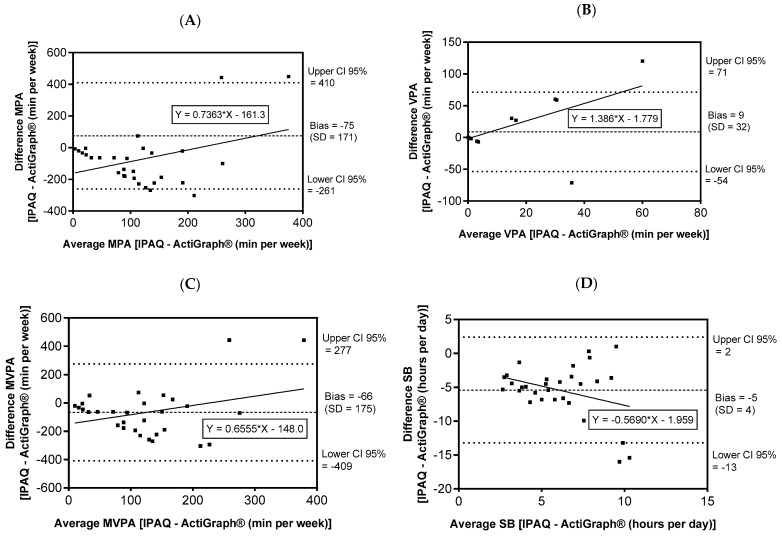
Bland–Altman graphs of people with major depressive disorder or bipolar disorder with measurements obtained by IPAQ and ActiGraph^®^. The x-axis (mean of the measurements obtained by IPAQ and ActiGraph^®^) and y-axis (difference in the measurements obtained by IPAQ and ActiGraph^®^). The bias, standard deviation (SD), and 95% confidence interval (CI) of the differences are presented as a linear regression (with an equation). Panel (**A**) = moderate physical activity (MPA); Panel (**B**) = vigorous physical activity (VPA); Panel (**C**) = moderate and vigorous physical activity (MVPA); Panel (**D**) = sedentary behavior (SB).

In Figure 3, we present the Bland-Altman graphs with the measurements obtained by the IPAQ and ActiGraph^®^ (pooled—MDD and BD together) for MPA (Panel A), VPA (Panel B), MVPA (Panel C), and SB (Panel D). 

For the PA variable, in Figure 3 Panel A, we found overestimation of the MPA data in the IPAQ relative to ActiGraph^®^, with poor agreement between the methods and a bias of −75 min (upper CI 410 min /lower CI −261 min). So, too, in Figure 3 Panel B, with overestimation of the VPA engagement times in the IPAQ relative to ActiGraph^®^, with poor agreement between the methods and a bias of 9 min (upper CI 71 min/lower CI −54 min).

In Figure 3 Panel C, we found an underestimation of MVPA engagement in the IPAQ relative to ActiGraph^®^, with poor agreement between the methods and a bias of −66 min (upper CI 277 min /lower CI −409 min). Finally, in Figure 3 Panel D, we found underestimation of SB engagement in the IPAQ relative to ActiGraph^®^, with poor agreement between the methods and a bias of −5 h (upper CI 2 h /lower CI −13 h). 

## 4. Discussion

To the best of our knowledge, our study is the first to demonstrate that PA time (MPA, VPA, and MVPA) and SB time in participants with MDD or BD, as assessed by the IPAQ or ActiGraph^®^, present (a) discrepant values in between-tool comparisons; (b) low associations; and (c) poor method agreement. These findings confirm our a priori hypothesis that PA and SB evaluated by subjective (IPAQ) and objective (ActiGraph^®^) instruments would show poor agreement. Additionally, MDD and BD, isolated or pooled, presented higher activity values for MPA, MVPA, and SB when measured with ActiGraph^®^ than when measured by self-reporting using the IPAQ; that is, the self-reported IPAQ scores underestimated the values of these measures relative to ActiGraph^®^. Below, we explore two principal points that can impact the meaning of the differences we observed: (a) details of the IPAQ questions; and (b) the characteristics of the respondents. 

As reported in our literature review, our findings of underestimates of PA and SB when comparing subjective and objective tools, respectively, seem to echo findings in several other studies [27,28,30]. Even though physical activity questionnaires need to be brief in order to reduce their administration time for participants, there may be limitations in the information provided and in the complete comprehension of these questions for respondents with mental disorders. Thus, it may be necessary to add more questions or details within these items so that people with MDD and BD better understand PA behaviors (e.g., considering different domains such as occupational PA, transport-related PA, and PA during discretionary or leisure time) and SB. For example, the IPAQ contains only a single question about SB: “During the last 7 days, how much time did you spend sitting?” This singular item may have contributed to the participants’ underestimating their SB relative to the ActiGraph^®^ results. If the IPAQ contained more items and details about the context of SB (e.g., time in bed overnight, sedentary time, time napping, time spent waking), there might have been a more accurate understanding of the differences between PA and SB and more accurate and precise self-reports that would more closely agree with the ActiGraph^®^ data. Another possibility for estimating PA and SB with better precision would be to use a questionnaire containing more questions and details about the behavior. Although the IPAQ is a long form with 27 questions, we highlight some limitations, as only two questions are related to SB, which is not sufficient to estimate SB with precision. Some researchers suggest using a diary to complement the information about PA and SB from the accelerometer, which could be valid; however, to our understanding, this is not a resolution for the IPAQ (especially because it is a retrospective questionnaire). Using a single or small number of questions about SB (such as within the short-form IPAQ or long-form IPAQ) has been shown to present poor validity for measuring SB, as evidenced when comparing other questionnaires with multiple items, such as the Marshall Sitting Time Questionnaire and Activity Questionnaire for Adults and Adolescents [44]. Questionnaires with multiple items assess a variety of types of SB, and consider different domains and days of the week and weekends separately [45], all of which can impact the total measured SB time (≌ more than 300 min).

Factors related to the questionnaire can impact the degree of agreement between these subjective and objective measures. Hallal et al. [46] previously called attention to these concerns about the IPAQ, on which each question involves actual activities carried out and PA and SB are inferred by combining questionnaire responses rather than by asking respondents directly about PA and SB. 

A recently introduced questionnaire for assessing PA and SB among patients with mental disorders, the Simple Physical Activity Questionnaire (SIMPAQ) [47], considers time spent in PA and SB and the context for these related questions. The SIMPAQ seeks information about time spent in bed overnight, sedentary time, including napping, time spent awake, time spent exercising, and time engaged in incidental activity, averaged over the previous seven-day period [47]. Furthermore, attempts to validate the SIMPAQ by comparing it to the accelerometer to measure PA showed good test–retest reliability [47]. These data support the idea that more detailed questionnaires are needed for self-report PA assessment, perhaps even more so for patients with mental disorders. 

A prior study with older adults [48] also suggested that it may be necessary to provide an example of a daily breakdown of typical activities performed to more accurately and precisely measure PA or SB through self-report. Other authors have mentioned a need for additional strategies when sampling self-reports of participants with severe mental illness [49], including the importance of the essential narrative synthesis about PA and SB underlying the investigator’s intent for using a specific questionnaire and whether the questionnaire choice was tied to how the study was first validated. This can be problematic if the tool was validated in a different population, as its psychometric properties may then differ from those necessary for its intended use with new respondents [49]. Firth and collaborators specifically warned that there may be inaccuracies when using the IPAQ for PA measurement with patients who have schizophrenia due to known differences between these participants and other populations with respect to relevant constructs such as diet, medication adherence, and social interactions [29]. Similarly, other studies with schizophrenia patients [30], Asian women [27], and a systematic review of 23 studies, using participants in the general population, participants with chronic diseases, and students [21], showed weak precision of the IPAQ as an indicator of PA.

Besides the configuration of questions within the IPAQ, the characteristics of IPAQ responders may influence the data gathered [23]. In the case of participants with MDD, impaired memory for positive events is common [24], and in BD, impaired recall and insight and concentration difficulties during periods of acute depression are common [50]. These characteristics may partly explain the poor agreement we observed between the IPAQ and ActiGraph^®^ for assessing PA and SB. Of importance, however, is that in the clinical environment, the use of the ActiGraph^®^ could be unworkable and, thus, the use of the IPAQ may be necessary, even if it is an inaccurate tool. Thus, there is a need for caution in interpretation of the results.

The assessment of PA and SB is of importance to the general health of individuals with MDD and BD, since sedentary lifestyle behaviors may negatively impact medical comorbidities [3,51]. However, our data and that of others, as reviewed here, suggest that health professionals should be cautious about the accuracy of PA and SB data in patients with MDD and BD if the data were gathered indirectly with the IPAQ. Finally, of relevance to earlier reports of more SB among persons with mental disorders, our participants performed 7 and 22 min of MVPA per week, based on IPAQ and ActiGraph^®^ data, respectively, while they performed 171 and 490 min of SB per day, based on IPAQ and ActiGraph^®^ data, respectively. By either measure, there is clearly a need to increase PA and reduce SB for this population [29], since physical inactivity has been shown to have a detrimental effect on health worldwide [52].

Our study presents specific limitations. First, our small sample size. Second, the ActiGraph model GT9-X has excellent sensitivity (90%) for walking, which adds important value to MPA, VPA, or MVPA data, but lower sensitivity for sitting and standing (57% and 53%) [53]. Finally, due to its ease of application and simplicity, we used the IPAQ in its short form to measure PA and SB, which does not allow us to generalize our findings to the longer IPAQ or other subjective instruments.

Future research should analyze MDD or BD populations considering a larger sample size. Questionnaires with more details about the behavior of PA or SB, for example, the three domains in which physical activity is performed (occupational physical activity, transport-related physical activity, and physical activity during discretionary or leisure time), such as the Global Physical Activity Questionnaire (GPAQ), could be used as a reference.

## 5. Conclusions

The current study adds new information to the literature regarding the poor agreement of PA and SB measured by the IPAQ and ActiGraph^®^ in participants diagnosed with mental disorders, specifically MDD or BD. Due to the differences observed between subjective (IPAQ) and objective (ActiGraph^®^) measurements, some caution should be adopted when interpreting subjective reports of PA and SB (using the IPAQ) in participants with MDD or BD. 

## Figures and Tables

**Table 1:** Sample characteristics.

Variable	MDD People (*n* = 18)	BD People (*n* = 12)
Female	83.3%	91.7%
Age (years)	42.5 ± 10.8	40.8 ± 14.2
Weight (kilograms)	82.8 ± 12.0	69.0 ± 16.0
Height (centimeters)	164 ± 11	162 ± 14
Body Mass Index (kg/m^2^)	30.6 ± 4.1	26.7 ± 5.6
Symptoms of disease		
Depression—MADRS (points)	14.7 ± 8.6	15.6 ± 9.1
Mania—YMRS (points)	---	7.2 ± 3.6
Marital status		
Single	50.0%	50.0%
Married	38.9%	33.3%
Divorced/Widowed	11.1%	16.7%
Schooling		
Elementary school	16.7%	33.3%
High school	38.9%	33.3%
University education	44.4%	33.3%
Profession or occupation		
Health and education	27.8%	8.3%
Housemaid	11.1%	8.3%
Unemployed and at home	33.3%	50.0%
Other areas	27.8%	33.3%
Comorbidities		
Hypertension	22.2%	25.0%
Diabetes	11.1%	8.3%
Obesity	61.1%	25.0%
Others	5.6%	8.3%
Number of drugs used for treating psychiatric disorders		
1 drug	33.3%	16.7%
2 drugs	50.0%	25.0%
>3 drugs	16.7%	58.3%
Subjective health perception		
Do you usually sleep well? (Yes)	38.9%	41.7%
Do you have a balanced diet? (Yes)	16.7%	16.7%
Do you smoke (tobacco/cigarette)? (Yes)	5.6%	33.3%
Do you use excessive alcohol? (Yes)	0%	0%
Do you perform systematic exercise? (Yes)	0%	16.7%

Age, weight, height, body mass index, and symptoms of depression and mania are expressed as the mean ± standard deviation. The other data are expressed as percentage (%). MDD = major depressive disorder; BD = bipolar disorder; kg = kilos; m² = square meter measurement. MADRS = Montgomery–Asberg Depression Rating Scale; YMRS = Young Mania Rating Scale.

**Table 2:** Comparison (Mann–Whitney test) of the measurements obtained by IPAQ (walking, MPA, VPA, MVPA, and SB) and ActiGraph^®^ (LPA, MPA, VPA, MVPA, and SB) of people with MDD (isolated), BD (isolated), and both (pooled—MDD and BD together).

Variable	MDD (*n* = 18)			BD (*n* = 12)			Pooled (*n* = 30)		
	ActiGraph^®^	IPAQ	*p*	ActiGraph^®^	IPAQ	*p*	ActiGraph^®^	IPAQ	*p*
Walking (Minutes per week)	--	39 (0–93)	--	--	73 (0–25)	--	--	40 (0–100)	
LPA (Minutes per week)	2221 (1637–2848)	--	--	2063 (1417–2368)	--	--	2193 (1568–2706)	--	--
MPA (Minutes per week)	167 (130–243)	25 (0–105)	**0.003**	113 (68–186)	28 (0–90)	**<0.001**	155 (76–222)	25 (0–110)	**<0.001**
VPA (Minutes per week)	0 (0–1)	0 (0–0)	ns	0 (0–1)	0 (0–0)	ns	0 (0–1)	0 (0–0)	ns
MVPA (Minutes per week)	167 (131–244)	45 (0–105)	**0.012**	114 (68–186)	50 (8–128)	ns	157 (76–223)	50 (0–120)	**0.005**
SB (Hours per day)	8 (6–9)	3 (2–6)	**<0.001**	8 (7–11)	3 (2–6)	**0.002**	8 (7–10)	3 (1–5)	**<0.001**

MDD = major depressive disorder; BD = bipolar disorder; LPA = light physical activity, MPA = moderate physical activity, VPA = vigorous physical activity, MVPA = moderate and vigorous physical activity, SB = sedentary behavior. Data expressed as medians and percentiles. ns = not significant.

## Data Availability

The datasets used and/or analyzed during the current study are available from the corresponding author on reasonable request.

## References

[1] American Psychiatric Association (2014). Diagnostic and Statistical Manual of Mental Disorders (DSM-5^®^).

[2] GBD (2020). Global burden of 369 diseases and injuries in 204 countries and territories, 1990–2019: A systematic analysis for the Global Burden of Disease Study 2019. Lancet.

[3] Firth J., Siddiqi N., Koyanagi A., Siskind D., Rosenbaum S., Galletly C., Allan S., Caneo C., Carney R., Carvalho A.F. (2019). The Lancet Psychiatry Commission: A blueprint for protecting physical health in people with mental illness. Lancet Psychiatry.

[4] Plana-Ripoll O., Pedersen C.B., Agerbo E., Holtz Y., Erlangsen A., Canudas-Romo V., Andersen P.K., Charlson F.J., Christensen M.K., Erskine H.E. (2019). A comprehensive analysis of mortality-related health metrics associated with mental disorders: A nationwide, register-based cohort study. Lancet.

[5] Caspersen C.J., Powell K.E., Christenson G.M. (1985). Physical activity, exercise, and physical fitness: Definitions and distinctions for health-related research. Public Health Rep..

[6] Schuch F.B., Stubbs B. (2019). The Role of Exercise in Preventing and Treating Depression. Curr. Sport. Med. Rep..

[7] Schuch F.B., Vancampfort D., Firth J., Rosenbaum S., Ward P.B., Silva E.S., Hallgren M., Ponce De Leon A., Dunn A.L., Deslandes A.C. (2018). Physical Activity and Incident Depression: A Meta-Analysis of Prospective Cohort Studies. Am. J. Psychiatry.

[8] Schuch F.B., Vancampfort D., Richards J., Rosenbaum S., Ward P.B., Stubbs B. (2016). Exercise as a treatment for depression: A meta-analysis adjusting for publication bias. J. Psychiatr. Res..

[9] Stubbs B., Vancampfort D., Firth J., Schuch F.B., Hallgren M., Smith L., Gardner B., Kahl K.G., Veronese N., Solmi M. (2018). Relationship between sedentary behavior and depression: A mediation analysis of influential factors across the lifespan among 42,469 people in low- and middle-income countries. J. Affect. Disord..

[10] Sun H.L., Gao X., Que X.M., Liu L., Ma J.S., He S.M., Gao Q., Wang T. (2020). The causal relationships of device-measured physical activity with bipolar disorder and schizophrenia in adults: A 2-Sample mendelian randomization study. J. Affect. Disord..

[11] Duarte C., Belizario G.O., Mathias K., Silva M., Roberto P., Greve J., Lafer B. (2020). Structured physical exercise in bipolar depression: A pilot study. Bipolar Disord..

[12] Melo M.C.A., Garcia R.F., de Araújo C.F.C., Rangel D.M., de Bruin P.F.C., de Bruin V.M.S. (2019). Physical activity as prognostic factor for bipolar disorder: An 18-month prospective study. J. Affect. Disord..

[13] Vancampfort D., Probst M., Wyckaert S., De Hert M., Stubbs B., Rosenbaum S., Sienaert P. (2016). Physical activity as a vital sign in patients with bipolar disorder. Psychiatry Res..

[14] De Sá Filho A.S., De Souza Moura A.M., Lamego M.K., Rocha N.B.F., Paes F., Oliveira A.C., Lattari E., Rimes R., Manochio J., Budde H. (2015). Potential therapeutic effects of physical exercise for bipolar disorder. CNS Neurol. Disord.-Drug Targets.

[15] SBRN (2012). Letter to the editor: Standardized use of the terms “sedentary” and “sedentary behaviours”. Appl. Physiol. Nutr. Metab. = Physiol. Appl. Nutr. Et Metab..

[16] Biswas A., Oh P.I., Faulkner G.E., Bajaj R.R., Silver M.A., Mitchell M.S., Alter D.A. (2015). Sedentary time and its association with risk for disease incidence, mortality, and hospitalization in adults: A systematic review and meta-analysis. Ann. Intern. Med..

[17] Kandola A.A., Del Pozo Cruz B., Osborn D.P.J., Stubbs B., Choi K.W., Hayes J.F. (2021). Impact of replacing sedentary behaviour with other movement behaviours on depression and anxiety symptoms: A prospective cohort study in the UK Biobank. BMC Med..

[18] Werneck A.O., Hoare E., Stubbs B., van Sluijs E.M.F., Corder K. (2021). Association of mentally-active and mentally-passive sedentary behaviour with depressive symptoms among adolescents. J. Affect. Disord..

[19] Vancampfort D., Firth J., Schuch F.B., Rosenbaum S., Mugisha J., Hallgren M., Probst M., Ward P.B., Gaughran F., De Hert M. (2017). Sedentary behavior and physical activity levels in people with schizophrenia, bipolar disorder and major depressive disorder: A global systematic review and meta-analysis. World Psychiatry Off. J. World Psychiatr. Assoc. (WPA).

[20] Craig C.L., Marshall A.L., Sjöström M., Bauman A.E., Booth M.L., Ainsworth B.E., Pratt M., Ekelund U., Yngve A., Sallis J.F. (2003). International physical activity questionnaire: 12-country reliability and validity. Med. Sci. Sport. Exerc..

[21] Lee P.H., Macfarlane D.J., Lam T.H., Stewart S.M. (2011). Validity of the International Physical Activity Questionnaire Short Form (IPAQ-SF): A systematic review. Int. J. Behav. Nutr. Phys. Act..

[22] Bauman A., Ainsworth B.E., Bull F., Craig C.L., Hagstromer M., Sallis J.F., Pratt M., Sjostrom M. (2009). Progress and pitfalls in the use of the International Physical Activity Questionnaire (IPAQ) for adult physical activity surveillance. J. Phys. Act. Health.

[23] Durante R., Ainsworth B.E. (1996). The recall of physical activity: Using a cognitive model of the question-answering process. Med. Sci. Sport. Exerc..

[24] Dillon D.G., Pizzagalli D.A. (2018). Mechanisms of Memory Disruption in Depression. Trends Neurosci..

[25] Walia H.K., Mehra R. (2019). Practical aspects of actigraphy and approaches in clinical and research domains. Handb. Clin. Neurol..

[26] Trost S.G., McIver K.L., Pate R.R. (2005). Conducting accelerometer-based activity assessments in field-based research. Med. Sci. Sport. Exerc..

[27] Curry W.B., Thompson J.L. (2015). Comparability of accelerometer- and IPAQ-derived physical activity and sedentary time in South Asian women: A cross-sectional study. Eur. J. Sport Sci..

[28] Pinto A.J., Roschel H., Benatti F.B., de Sá Pinto A.L., Sallum A.M., Silva C.A., Gualano B. (2016). Poor agreement of objectively measured and self-reported physical activity in juvenile dermatomyositis and juvenile systemic lupus erythematosus. Clin. Rheumatol..

[29] Firth J., Stubbs B., Vancampfort D., Schuch F.B., Rosenbaum S., Ward P.B., Firth J.A., Sarris J., Yung A.R. (2018). The Validity and Value of Self-reported Physical Activity and Accelerometry in People With Schizophrenia: A Population-Scale Study of the UK Biobank. Schizophr. Bull..

[30] Duncan M.J., Arbour-Nicitopoulos K., Subramanieapillai M., Remington G., Faulkner G. (2017). Revisiting the International Physical Activity Questionnaire (IPAQ): Assessing physical activity among individuals with schizophrenia. Schizophr. Res..

[31] Amorim P. (2000). Mini International Neuropsychiatric Interview (MINI): Validação de entrevista breve para diagnóstico de transtornos mentais. Rev. Bras. Psiquiatr..

[32] Sheehan D.V., Lecrubier Y., Sheehan K.H., Amorim P., Janavs J., Weiller E., Hergueta T., Baker R., Dunbar G.C. (1998). The Mini-International Neuropsychiatric Interview (MINI): The development and validation of a structured diagnostic psychiatric interview for DSM-IV and ICD-10. J. Clin. Psychiatry.

[33] Goodyear M.D., Krleza-Jeric K., Lemmens T. (2007). The declaration of Helsinki. BMJ.

[34] Montgomery S.A., Asberg M. (1979). A new depression scale designed to be sensitive to change. Br. J. Psychiatry.

[35] Davidson J., Turnbull C.D., Strickland R., Miller R., Graves K. (1986). The Montgomery-Åsberg Depression Scale: Reliability and validity. Acta Psychiatr. Scand..

[36] Young R.C., Biggs J.T., Ziegler V.E., Meyer D.A. (1978). A rating scale for mania: Reliability, validity and sensitivity. Br. J. Psychiatry J. Ment. Sci..

[37] Vilela J., Crippa J.A.d.S., Del-Ben C.M., Loureiro S.R. (2005). Reliability and validity of a Portuguese version of the Young Mania Rating Scale. Braz. J. Med. Biol. Res..

[38] Sasaki J., Coutinho A., Santos C., Bertuol C., Minatto G., Berria J., Tonosaki L., Lima L., Marchesan M., Silveira P. (2017). Orientações para utilização de acelerômetros no Brasil. Rev. Bras. De Ativ. Física Saúde.

[39] Choi L., Liu Z., Matthews C.E., Buchowski M.S. (2011). Validation of accelerometer wear and nonwear time classification algorithm. Med. Sci. Sport. Exerc..

[40] Sasaki J.E., John D., Freedson P.S. (2011). Validation and comparison of ActiGraph activity monitors. J. Sci. Med. Sport.

[41] Schober P., Boer C., Schwarte L.A. (2018). Correlation coefficients: Appropriate use and interpretation. Anesth. Analg..

[42] Mansournia M.A., Waters R., Nazemipour M., Bland M., Altman D.G. (2021). Bland-Altman methods for comparing methods of measurement and response to criticisms. Glob. Epidemiol..

[43] Bland J.M., Altman D.G. (2010). Statistical methods for assessing agreement between two methods of clinical measurement. Int. J. Nurs. Stud..

[44] Prince S.A., LeBlanc A.G., Colley R.C., Saunders T.J. (2017). Measurement of sedentary behaviour in population health surveys: A review and recommendations. PeerJ.

[45] Healy G.N., Clark B.K., Winkler E.A., Gardiner P.A., Brown W.J., Matthews C.E. (2011). Measurement of adults’ sedentary time in population-based studies. Am. J. Prev. Med..

[46] Hallal P.C., Gomez L.F., Parra D.C., Lobelo F., Mosquera J., Florindo A.A., Sarmiento O.L. (2010). Lessons learned after 10 years of IPAQ use in Brazil and Colombia. J. Phys. Act. Health.

[47] Rosenbaum S., Morell R., Abdel-Baki A., Ahmadpanah M., Anilkumar T.V., Baie L., Bauman A., Bender S., Boyan Han J., Brand S. (2020). Assessing physical activity in people with mental illness: 23-country reliability and validity of the simple physical activity questionnaire (SIMPAQ). BMC Psychiatry.

[48] Cleland C., Ferguson S., Ellis G., Hunter R.F. (2018). Validity of the International Physical Activity Questionnaire (IPAQ) for assessing moderate-to-vigorous physical activity and sedentary behaviour of older adults in the United Kingdom. BMC Med. Res. Methodol..

[49] Soundy A., Roskell C., Stubbs B., Vancampfort D. (2014). Selection, use and psychometric properties of physical activity measures to assess individuals with severe mental illness: A narrative synthesis. Arch. Psychiatr. Nurs..

[50] Ravindran A.V., Balneaves L.G., Faulkner G., Ortiz A., McIntosh D., Morehouse R.L., Ravindran L., Yatham L.N., Kennedy S.H., Lam R.W. (2016). Canadian Network for Mood and Anxiety Treatments (CANMAT) 2016 Clinical Guidelines for the Management of Adults with Major Depressive Disorder: Section 5. Complementary and Alternative Medicine Treatments. Canadian journal of psychiatry. Rev. Can. De Psychiatr..

[51] Stubbs B., Vancampfort D., Hallgren M., Firth J., Veronese N., Solmi M., Brand S., Cordes J., Malchow B., Gerber M. (2018). EPA guidance on physical activity as a treatment for severe mental illness: A meta-review of the evidence and Position Statement from the European Psychiatric Association (EPA), supported by the International Organization of Physical Therapists in Mental Health (IOPTMH). Eur. Psychiatry.

[52] Bull F.C., Al-Ansari S.S., Biddle S., Borodulin K., Buman M.P. (2020). World Health Organization 2020 guidelines on physical activity and sedentary behaviour. Br. J. Sports Med..

[53] Valkenet K., Veenhof C. (2019). Validity of three accelerometers to investigate lying, sitting, standing and walking. PLoS ONE.

